# Cyanobacteria *Microcystis aeruginosa* Contributes to the Severity of Fish Diseases: A Study on Spring Viraemia of Carp

**DOI:** 10.3390/toxins13090601

**Published:** 2021-08-28

**Authors:** Miroslava Palikova, Radovan Kopp, Jiri Kohoutek, Ludek Blaha, Jan Mares, Petra Ondrackova, Ivana Papezikova, Hana Minarova, Lubomir Pojezdal, Ondrej Adamovsky

**Affiliations:** 1 Department of Ecology and Diseases of Zoo Animals, Game, Fish and Bees, Faculty of Veterinary Hygiene and Ecology, University of Veterinary Sciences Brno, 61242 Brno, Czech Republic; palikovam@vfu.cz (M.P.); ivana.papezikova@mendelu.cz (I.P.); minarovah@vfu.cz (H.M.); 2 Department of Zoology, Fisheries, Hydrobiology and Apiculture, Faculty of Agronomy, Mendel University in Brno, 61300 Brno, Czech Republic; fcela@seznam.cz (R.K.); jan.mares@mendelu.cz (J.M.); 3 RECETOX (Research Centre for Toxic Compounds in the Environment), Faculty of Science, Masaryk University, 62500 Brno, Czech Republic; jiri.kohoutek@recetox.muni.cz (J.K.); blaha@recetox.muni.cz (L.B.); 4 Department of Infectious Diseases and Preventive Medicine, Veterinary Research Institute, 62100 Brno, Czech Republic; petra.ondrackova@vri.cz (P.O.); lubomir.pojezdal@vri.cz (L.P.)

**Keywords:** cyanobacteria, spring viraemia of carp, microcystins, conjugates, immune system

## Abstract

Fish are exposed to numerous stressors in the environment including pollution, bacterial and viral agents, and toxic substances. Our study with common carps leveraged an integrated approach (i.e., histology, biochemical and hematological measurements, and analytical chemistry) to understand how cyanobacteria interfere with the impact of a model viral agent, *Carp sprivivirus* (SVCV), on fish. In addition to the specific effects of a single stressor (SVCV or cyanobacteria), the combination of both stressors worsens markers related to the immune system and liver health. Solely combined exposure resulted in the rise in the production of immunoglobulins, changes in glucose and cholesterol levels, and an elevated marker of impaired liver, alanine aminotransferase (ALT). Analytical determination of the cyanobacterial toxin microcystin-LR (MC-LR) and its structurally similar congener MC-RR and their conjugates showed that SVCV affects neither the levels of MC in the liver nor the detoxification capacity of the liver. MC-LR and MC-RR were depurated from liver mostly in the form of cysteine conjugates (MC-LR-Cys, MC-RR-Cys) in comparison to glutathione conjugates (LR-GSH, RR-GSH). Our study brought new evidence that cyanobacteria worsen the effect of viral agents. Such inclusion of multiple stressor concept helps us to understand how and to what extent the relevant environmental stressors co-influence the health of the fish population.

## 1. Introduction

In the environment, specifically in eutrophic waters, fish are exposed to numerous stressors including increased temperature, pollutants, toxic cyanobacteria, and pathogens. While we can analyze the chemical and physical parameters of the aquatic environment and the level of pollution, we poorly understand how biological agents, specifically in cases when they coexist in a single environment, may affect the health of aquatic animals, including fish. Fish viruses, such as *Carp sprivivirus* (formerly spring viraemia of carp virus, SVCV), are integral parts of the environment and responsible for fish mortality and morbidity [1]. Spring viraemia of carp (SVC) is a widespread disease in European carp culture affecting cyprinids, mainly farmed common carp (*Cyprinus carpio* L.). In addition, increasing levels of nutrients and the rise in global temperature result in dramatic growth of other biological agents—the cyanobacteria [2]. According to the United States Environmental Protection Agency (US EPA), toxic species of cyanobacteria represent one of the most serious environmental problems for aquatic and human health worldwide [3]. Cyanobacteria produce a large spectrum of toxic substances that were shown to affect aquatic animals from crustaceans, fish, and birds to mammals [4,5]. Despite the number of structurally different toxins produced by cyanobacteria, one of the most studied cyanobacterial toxins are hepatotoxic microcystins (MCs). Although an individual stressor present in the environment may not represent a significant risk for aquatic organisms, a combination of multiple factors can produce unforeseen effects, especially if they target the same biological system or function (e.g., immune system). Despite the immunomodulatory potency of cyanobacterial toxins being confirmed both in vitro and in vivo [6,7,8,9], data on the cyanobacterial influence on the manifestation of diseases or on the susceptibility of individuals to bacterial and viral agents frequently present in the environment together with cyanobacteria, are sparse. A limited number of studies indicate that cyanobacteria may negatively contribute to the effects of other stressors.

For example, our previous study with common carps exposed to white spot disease and/or cyanobacterial biomass showed that exposure to a single stressor did not significantly impact the immune system; nevertheless, the combination of both stressors caused immunosuppression, specifically inhibition of the oxidative burst of fish phagocytes [10]. In this study, cyanobacteria were shown to have not only a strong impact on the nonspecific immune response in fish but contributed to the increased susceptibility to infectious diseases. In addition, recent studies with rodents indicate that cyanobacteria and their toxins are significant factors exacerbating diseases. In a study with mice, MCs affected the severity of diet-induced nonalcoholic steatohepatitis (NASH) [11], and they also exacerbated hepatic injury and intestinal inflammation in a murine model of nonalcoholic fatty liver disease (NAFLD) [12,13,14]. However, cyanobacterial toxins may worsen existing diseases; the effect of cyanobacteria specifically on fish diseases has not been studied so far in detail.

The aim of our study was to investigate the influence of cyanobacterial biomass and infection agent represented by the *Carp sprivivirus* (SVCV), as both are commonly present in aquatic environments and affect fish health. The scientific objective was to determine how and to what extent cyanobacteria contributes to the effects of SVCV in fish. To fully understand the impact of both stressors, we investigated a series of clinical signs, mortality, biochemical, and hematological indicators, histopathology, and toxin accumulation and depuration.

## 2. Results

### 2.1. Body Size Measurements

The following parameters were evaluated at T0, T7, T14, T21, T28, and T42: the length of the body, height and width of the body, weight of the body without entrails, and weight of the liver. No significant changes were found between the exposed groups and control group (data not shown).

### 2.2. Hematology

**Figure 1:**
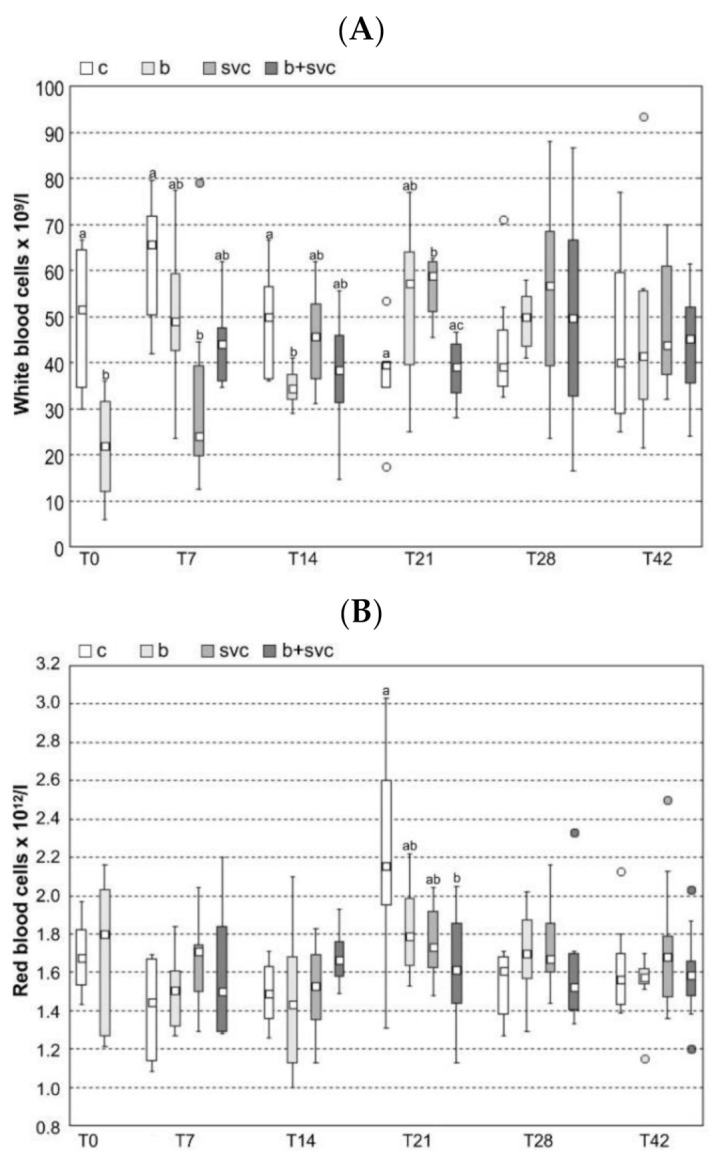

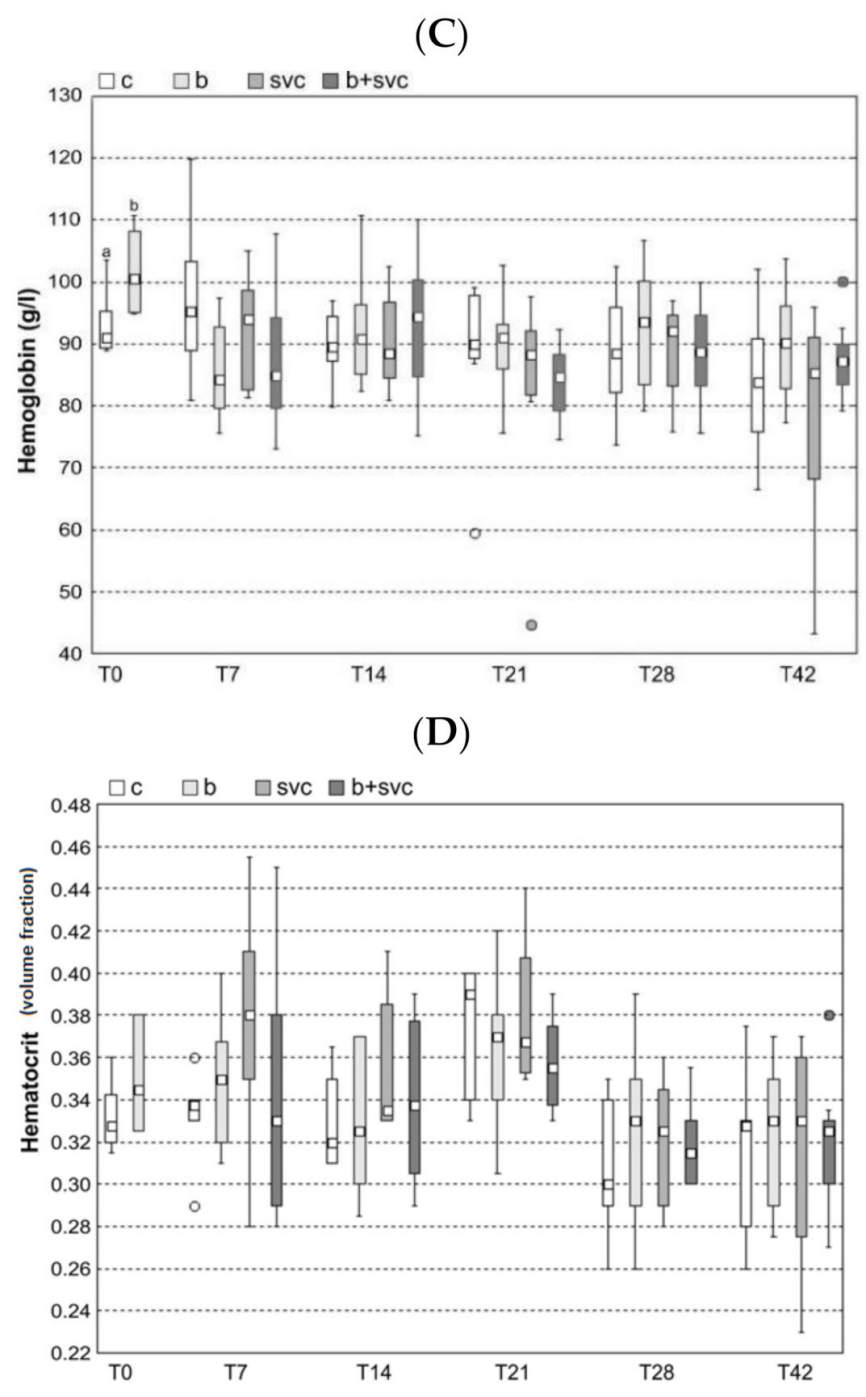
Hematology ((**A**) total white blood cell count, (**B**) total red blood cell count, (**C**) hemoglobin and (**D**) hematocrit) in fish exposed to cyanobacterial biomass, spring viremia of carp, or both cyanobacterial biomass and spring viremia of carp. *Carp sprivivirus* was applied seven days after the start of cyanobacterial biomass exposition. This day was assigned as day 0 (T0). The sampling of eight fish from each group (N = 8) was performed at days 0, 7, 14, 21, 28, and 42 (i.e., T0, T7, T14, T21, T28, T42). Box includes the 25th to 75th percentiles, with the middle point representing the median and the spots showing the outliers. c—control, b—cyanobacterial biomass exposed group, svc—spring viremia of carp infected group, B+SVC—combined exposure to cyanobacterial biomass and spring viremia of carp, T—days after start of the experiment. Significant differences among indices are marked by lower letters (*p* < 0.05) or capital letters (*p* < 0.01).

The hematological parameters were measured in all groups at T0, T7, T14, T21, T28, and T42 (Figure 1). The count of white blood cells (Figure 1A) was decreased in group B (T0), groups SVC and B+SVC (T7), and group B (T14) compared to group C. On the other hand, the count of white blood cells was found to be increased in group SVC (T21) compared to groups C and B+SVC. Monocyte counts were increased in group B+SVC (T7), in groups SVC and B+SVC (T21), and in groups SVC and B+SVC compared to group B (T14) (Figure S1). Neutrophil counts were significantly increased in group B in T28 (Figure S1). The count of red blood cells was decreased in group B+SVC (T14) compared to group C (Figure 1B). Hemoglobin was increased in group B (T0) compared to group C (Figure 1C). There were no significant changes in hematocrit among groups (Figure 1D). Blast cells were significantly increased in group B+SVC in T14 (Figure S1). Phagocyte numbers were increased in group SVC (T21) and group B (T28) (Figure S1). The lymphocyte/phagocyte ratio was decreased in groups SVC and B+SVC (T7), in group B+SVC (T14), in groups SVC and B+SVC (T21), and in groups B and B+SVC (T28) (Figure S1). Lymphocytes were decreased in group B (T0), in groups SVC and B+SVC (T7), in group B (T14), and in group B+SVC (T21) (Figure S1). Monocyte numbers were increased in group B+SVC (T7) and in groups SVC and B+SVC (T14). In T21, monocytes were increased in group SVC compared to control and group B, and in group B+SVC compared to control (Figure S1). Myelocyte numbers were increased in group B (T28). Segmented neutrophils were increased in group SVC compared to group B (T21) (Figure S1).

### 2.3. Total Immunoglobulin Level

**Figure 2:**
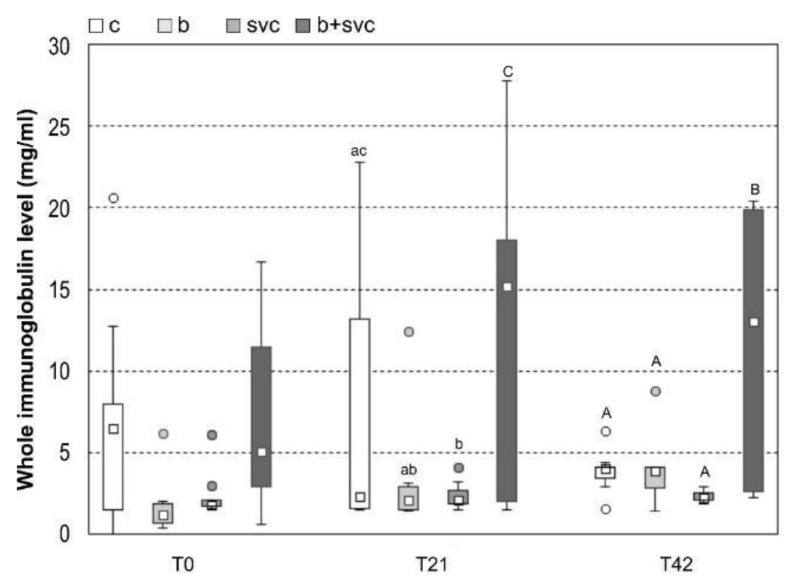
The whole immunoglobulin level measured by ELISA. Ten fish from each group were exposed to cyanobacterial biomass (B) or *Carp sprivivirus* (SVCV) or both cyanobacterial biomass and spring viremia of carp (B+SVC). *Carp sprivivirus* was applied seven days after the start of cyanobacterial biomass exposition. This day was assigned as day 0 (T0). The sampling was performed at days 0, 21, and 42 (i.e., T0, T21, T42). Box includes the 25th to 75th percentiles, with the middle point representing the median and the spots showing the outliers. Significant differences among indices are marked by lower letters (*p* < 0.05) or capital letters (*p* < 0.01).

Total immunoglobulin level was measured in ten fish from all groups at T0, T21, and T42 by ELISA. The total immunoglobulin level was significantly higher at group B+SVC at T21 and T42 compared to all other groups. Moreover, the level was significantly increased at group B+SVC from T0 to T21 and from T0 to T42. Furthermore, the level significantly decreased in group SVC at T21 (Figure 2).

### 2.4. Respiratory Burst

**Figure 3:**
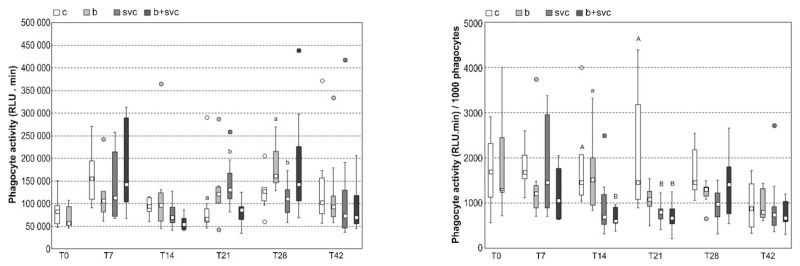
Respiratory burst. Data represent integrals of kinetic curves: chemiluminescence response of zymosan-stimulated blood in relative light units (RLU) x min, and chemiluminescence response expressed as signal per 1000 phagocytes—RLU x min/1000 phagocytes) in eight fish from each group exposed to cyanobacterial biomass or spring viremia of carp or both (B+SVC). Virus of SVC was applied seven days after the start of cyanobacterial biomass exposition. This day was assigned as day 0 (T0). Box plot is 25th to 75th percentiles, with the middle point representing the median and the spots showing the outliers. Groups: c—control, b—cyanobacterial biomass exposed group, svc—spring viremia of carp infected group, B+SVC—combined group. Significant differences among indices are marked by lower letters (*p* < 0.05) or capital letters (*p* < 0.01).

The chemiluminescence response of zymosan-stimulated blood was measured in all groups at T0–T42 by luminometry and was also expressed as a signal per 1000 phagocytes present in the sample (Figure 3). The chemiluminescence response of zymosan-stimulated blood was significantly increased in group SVC (T21) compared to the control group. Chemiluminescence response per 1000 phagocytes was significantly decreased in group B+SVC (T14) and in groups SVC and B+SVC (T21). The stimulation index (ratio of chemiluminescence response of stimulated and non-stimulated blood) was decreased in group SVC (T28) compared to group B (T28) (Figure S2).

### 2.5. Blood Biochemistry

**Figure 4:**
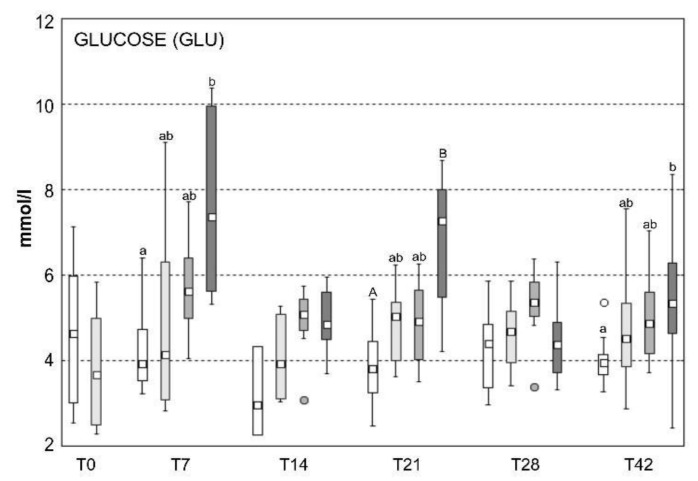
The level of glucose in blood plasma of common carp (N = 8 per group). Box includes the 25th to 75th percentiles, with the middle point representing the median and the spots showing the outliers. c—control group, b—cyanobacterial biomass exposed group, svc—spring viremia of carp infected group, B+SVC—combined (B+SVC) group, T—days after start of the experiment. Significant differences among indices are marked by lower letters (*p* < 0.05) or capital letters (*p* < 0.01).

The statistically significant changes of biochemical analyses of blood parameters are summarized in Table 1. Levels of glucose (GLU) are presented in Figure 4. Other statistically significant parameters are visualized in the Supplementary Materials (Figure S3). The levels of aspartate aminotransferase (AST), lactate (LACT), triglycerides (TRIG), and electrolytes (Na, K, Cl) were not statistically affected by any of the treatments.

### 2.6. Pathological Evaluation and Histopathology

Ten days after the SVCV application, one fish from the B+SVC group died. Exophthalmos, hemorrhages in the skin, yellowish fluid in body cavity, and hepatopancreas with yellow–green color were found during post-mortem examination. Pathological changes were found in the SVC and B+SVC groups at T7 and T14. Pathological changes were generally expressed as the presence of petechial hemorrhages on the ventral part of the body (SVC T7: *n* = 6, B+SVC T7: *n* = 3, SVC T14: *n* = 4, B+SVC T14: *n* = 1) and in the gut (SVC T7: *n* = 1, B+SVC T7: *n* = 5). At later time points, the fish recovered, and no further pathological changes were apparent anymore.

**Figure 5:**
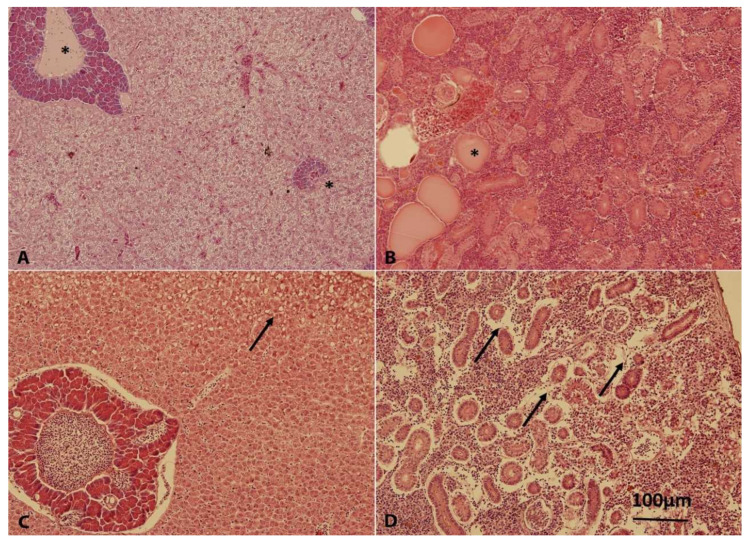
Generalized glycogen hepatodystrophy, intact islets of pancreatic glands (*): SVC, T14 (**A**), cystic formations with homogenous eosinophilic substance in the kidney parenchyma (*): B+SVC, T14 (**B**), focal macrovesicular fatty dystrophy of liver (→): B, T48 (**C**), peritubular oedema of kidney (→): B, T48 (**D**). H&E staining, magnification 200x.

By histological examination, hepatodystrophy and the presence of cystic formations with homogenous eosinophilic substances in the kidney parenchyma were observed (Figure 5). Sporadically, mononuclear infiltration in the stroma of intestinal villi were observed in groups B, SVC, and B+SVC in T14. However, except for the more frequent presence of cystic formation, no differences were observed between groups. In T42, focal macrovesicular fatty dystrophy of liver and peritubular and perivascular edema and the presence of clusters of inflammation cellular events in the kidney were observed in all of the above-mentioned groups. The presence of cystic formation was observed only sporadically (Figure 5).

### 2.7. Burden of Microcystins in the Liver

**Figure 6:**
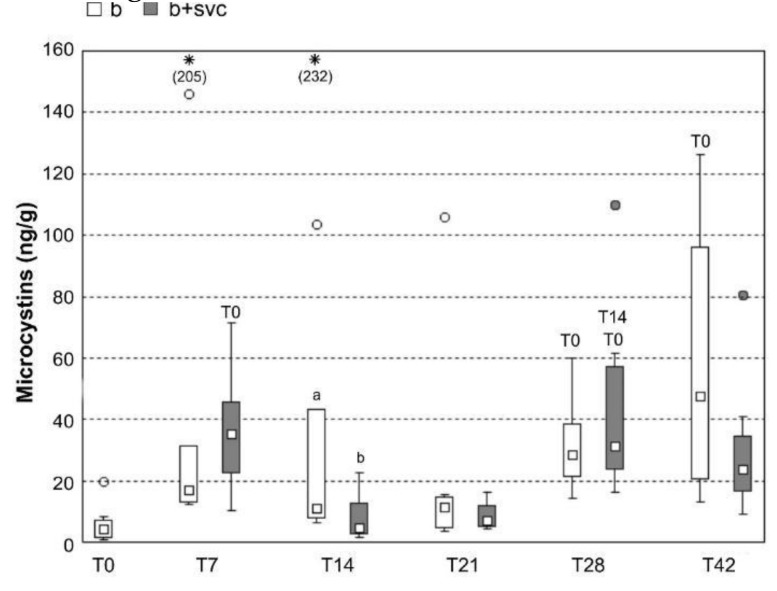
Concentration of MCs (MC-LR+MC-RR) in liver of common carp exposed to cyanobacterial biomass (alone or in combination with virus) in different sampling points (T0–T42 in days). Box plots include the 25th to 75th percentiles, with the middle point representing the median, the spots showing the outliers and asterisk the extremes. B—cyanobacterial biomass exposed group, B+SVC – combined exposure to cyanobacterial biomass and spring viremia of carp, T—days after start of the experiment. Significant differences among indices are marked by lower letters (*p* < 0.05) or capital letters (*p* < 0.01). Eight fish from each group were tested in each time point. Statistical differences between time points are labeled with time points (e.g., T14) to which we found a statistical significant differences.

The concentration of MCs in the liver was measured at groups B and B+SVC at T0–T42 (Figure 6). The concentration of MCs in the liver was significantly lower in group B+SVC (T14) compared to group B. The concentration of MCs in group B as well as B+SVC changed significantly during the time. The concentration was significantly increased in group B as well as in group B+SVC at T7, T28, and T42 compared to T0. On the other hand, a transient significant decrease in MC concentration occurred in group B+SVC only at T14.

### 2.8. Concentration of Microcystin Conjugates in the Liver

**Figure 7:**
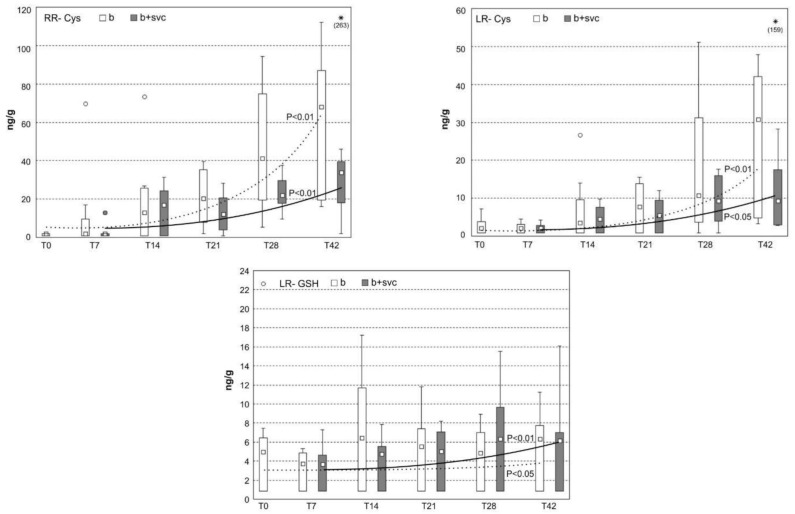
Concentration of microcystin conjugates RR-Cys, LR-Cys, and LR-GSH in fish liver (*n* = 8 per group). The fish were exposed to cyanobacterial biomass (group b) or both cyanobacterial biomass and spring viremia of carp (B+SVC group). SVC was applied seven days after the start of cyanobacterial biomass exposition. This day was assigned as day 0 (T0). The sampling was performed at days 0, 7, 14, 21, 28, and 42 (i.e., T0, T7, T14, T21, T28, and T42). Box includes the 25th to 75th percentiles, with the middle point representing the median, the spots showing the outliers, and the asterisk showing the extremes. Markers of statistical significance (e.g., *p* < 0.01) show the statistically significant increase in conjugates with time as evaluated by the regression curve (dotted for group “b” and full line for group B+SVC).

The concentration of MC conjugates in the liver was measured in groups B and B+SVC at T0–T42 (Figure 7). There were no statistical difference in the levels of RR–cystein and LR–cystein conjugates (RR-Cys, LR-Cys) and glutathione conjugates (RR-GSH, LR-GSH) between groups in each time point. The levels of RR-Cys, LR-Cys, and LR-GSH significantly increased during the time in group B as well as in group B+SVC. The concentration of RR-GSH conjugates was mostly below or close to the detection limit of the method; thus, no statistical evaluation was performed for RR-GSH.

### 2.9. Level of Specific Antibodies against SVCV

The level of specific antibodies against SVCV was measured in ten fish per group in groups SVC and B+SVC at T0, T21, and T42 by ELISA. The level was found to be zero at T0 (day of infection), and it significantly increased at T21 and at T42. The titer of antibodies in groups SVC and B+SVC were 1720 ± 1044 and 1700 ± 1071, respectively, while for T21 and T42, they were 2200 ± 1746 and 2320 ± 1657, respectively. Although the level of antibodies appeared to be slightly increased from T21 to T42, the difference was not significant. In conclusion, there were no differences in the level of antibodies against SVCV between groups.

## 3. Discussion

Cyanobacteria and SVCV are integral parts of the aquatic habitat and are known for their ability to affect fish health. Our study indicates that both cyanobacteria and SVC modulate the number of white blood cells in fish (WBC, Figure 1). The effect of SVC on WBC was expected, as the known manifestation of SVC is an initial decrease in lymphocytes [1]. In contrast, long-term SVCV infection leads to an increase in neutrophils, monocytes, eosinophils, and basophils in fish [1]. This is in accordance with our results, which show a significant decrease in lymphocyte counts and an increase in monocyte and neutrophil counts (Figure S1). Furthermore, our results show the SVC-dependent decrease in WBC in the first week, which is followed by an increase in WBC in T21. In addition to fish exposed to SVCV, the fish exposed to complex cyanobacteria biomass had also decreased WBC in weeks T0 and T14 (Figure 1). Cyanobacterial biomass is a complex mixture of bioactive substances, and only a fraction of these substances were studied for their potency to interfere with the immune system. In addition to the potential role of cyanobacterial lipopolysaccharides (LPS) [15], the increasing amount of evidence indicates that the most abundant group of cyanobacterial toxins, microcystins (MCs), may play a role in the observed immune-related effects. For example, a study with mice reported that prolonged exposure to MCs can simultaneously decrease T and B cells in vivo [16]. Similarly, MCs or cyanobacterial biomass in the diet resulted in the decrease in specific subpopulations of lymphocytes in a study with rats [9]. Nevertheless, the specific mechanism of how MCs deregulate immune cells, or how these effects are triggered, is still not known in detail. Interestingly, the cyanobacteria related decrease in WBC did not impact the whole immunoglobulin level (Figure 2), indicating that cyanobacteria do not stimulate or inhibit the production of immunoglobulins.

Respiratory burst is a general term for the release of reactive oxygen species (ROS) by several types of immune cells implicated in the degradation of pathogens and cell signaling. Despite the association of SVC with a rise of neutrophil and monocyte numbers (Figure S1), respiratory burst intensity was increased only in the SVC group at T21 (Figure 3). When the results were expressed as chemiluminescence signal per 1000 phagocytes to evaluate the metabolic activity of phagocytes independently on their blood counts, it was found that the response of phagocytes to zymosan was significantly suppressed in the B+SVC group (T14) and in the SVC and B+SVC groups (T21) (Figure S2). An in vitro study of Rymuszka et al. (2010) working with defined numbers of isolated carp phagocytes and with several concentrations of MC-LR showed that low microcystin concentrations stimulated the respiratory burst of phagocytes, while the exposure to high concentrations led to the suppression of phagocyte metabolic activity reflected by a decrease in superoxide production during respiratory burst [17]. Similar results were obtained on phagocytes isolated from rainbow trout head kidney [18]. In our work, the decrease in phagocyte metabolic activity was probably counterbalanced by increased neutrophil and monocyte counts, leading to mostly non-significant changes in zymosan-stimulated respiratory burst.

Although previous studies show that both SVCV and cyanobacteria deregulate the immune system [6,7,8], we observed that the combination of stressors (B+SVC) has unique characteristics and impact on immunity. Specifically, in comparison to control and effects of individual stressors, solely combined exposure to B+SVC resulted in a dramatic rise in the production of immunoglobulins (Figure 2). In addition, the combined exposure to B+SVC systematically elevated blood glucose levels. B+SVC combined exposure was the only group that significantly differed from the control group in time points T7, T21, and T42 (Figure 4). Infections, as well as other immune-related stressors, can raise blood glucose via the modulation of various hormone-regulated pathways [19]. Furthermore, the elevated level of immunoglobulins in group B+SVC may indicate the activation of B cells that have increased energy requirements and, in case of activation, switch to a more anabolic metabolism compared to their naïve counterparts [19]. High level of glucose also reflect an increased glycolysis and oxidative phosphorylation, as activated B cells increase glucose uptake as a response to an increased energy demand that deals with the production of immunoglobulins [20]. Additionally, the combined exposure may deregulate hepatopancreas and metabolism, as both stressors strongly affect the level of glucose in blood and markers of health of hepatopancreas.

The combined exposure (B+SVC) was also the only exposure that was able to elevate an important marker of impaired hepatopancreas, ALT (T21, Table 1 and Figure S3). The level of ALT is commonly measured clinically as a biomarker for liver health; specifically, ALT is released from impaired liver to blood and serves as an early marker of liver diseases. Abnormal ALT levels can signify a severe underlying medical condition, which is typically related to the liver, bones, or kidney [21]. The adverse outcome of the long-term exposure to cyanobacterial biomass and SVC was also confirmed as pathological changes in the liver (i.e., hepatodystrophy) and in the kidney parenchyma (Figure 5). Liver was identified as a target organ also in studies where fish, birds, and mammals were exposed to microcystin-rich cyanobacterial biomass [22,23,24,25]. The published liver pathologies included necrosis, steatosis of the liver parenchyma, and vacuolar and granular dystrophy. Liver and the production of cholesterol are tightly connected. Generally, liver cells contribute to the conversion of fat into cholesterol and release cholesterol into the bloodstream. Various changes in cholesterol metabolism can be indicators of hepatic and biliary dysfunction [26]. Interestingly, combined stressors (B+SVC) increased cholesterol levels in blood (T21, T42; Table 1 and Figure S3), suggesting that the deregulation of lipid metabolism occurs only when both stressors are combined. Other analyzed biochemical and hematological parameters (i.e., alkaline phosphatase, creatine, phosphorus, lactate dehydrogenase, magnesium, total protein, urea, and calcium) were not systematically modulated by any of the experimental conditions (Table 1 and Figure S3).

SVCV is known for its potency to induce the pathology of hepatopancreas in fish, more specifically multifocal necrosis, adipose degeneration, and hyperemia [27]. Additionally, microcystins (MCs), known as hepatotoxic peptides, also target hepatocytes and induce liver damage in various animals, including fish [28]. As both stressors target liver cells, it is of interest to investigate how viral infection affects toxicokinetics and depuration machinery of hepatotoxic MCs in fish. In agreement with other studies showing the ability of MCs to accumulate in the liver [29], both MC variants (MC-LR and MC-RR) were detected in fish hepatopancreas in every studied time point (Figure 6 and Figure S4). MCs are transported to hepatocytes due to expression of the specific set of cell transmembrane transporters known as organic-anion-transporting polypeptides (e.g., OATPs 1B1, 1B3) [30]. The potency to enter the cell significantly affects not only the concentration of MCs and their conjugates in liver cells but also the toxicity [31]. Although MC-LR has generally higher affinity to OATPs, the higher concentration of MC-RR in the hepatopancreas may be a result of the higher dose of MC-RR (Figure 6). The cyanobacterial biomass used in our study contained a higher portion of MC-RR (57%) than MC-LR (43%). To more deeply investigate the impact of SVC on the depuration capacity of the fish hepatopancreas, MC conjugates that are able to eliminate MCs were analyzed (Figure 7). MCs are intensively depurated from the liver by enzymatically formed MC glutathione and cysteine conjugates via glutathione S-transferase (sGST) enzyme [32]. We identified that cysteine conjugates (LR-Cys, RR-Cys) are the dominant metabolites in comparison to glutathione conjugates (LR-GSH, RR-GSH), as the concentration of Cys conjugates are an order of magnitude higher then GSH conjugates. The detoxification of MC is a rapid and efficient process since the content of MC-Cys conjugates in hepatopancreas are in similar or higher levels than their free types, and the conjugates were identified shortly after the exposure (Figure 7). The observed high level of MC-Cys conjugates and lack or low level of MC-GSH conjugates were previously reported in several other studies. For example, in the study with bighead carp, where MCs were i.p. injected, MC-LR and MC-RR were excreted mostly in the form of MC-LR/RR-Cys rather than MC-LR/RR-GSH [33]. The study assumes that MCs-GSH might act as mid-metabolites and are changed to the more stable MCs-Cys rapidly [33]. In agreement with a study with bighead carp, it was described that MC-GSH conjugates act as a highly reactive intermediate and are rapidly converted to MC-Cys conjugates [34]. Our results also indicate that co-exposure with SVC may affect the level of both free MCs as well as MC-Cys conjugates, as they are systematically decreased in all time points in the combined exposure group. MCs are known as inducers of oxidative stress in the liver [35], where Cys mediates protection against reactive molecules produced by ROS [36,37]. The decrease in Cys conjugates in the co-exposed group (B+SVC) may reflect the enhanced need of Cys for both MC-induced detoxification processes connected with ROS production and the elimination of ROS generated from an SVC-activated immune system [38]. The potency of SVC to modulate the detoxification could be confirmed in follow-up study using a high number of tested individuals.

In conclusion, we leverage an integrated approach (i.e., histology, biochemical, and hematological measurements and analytical chemistry) to understand the potency of an environmentally relevant stressor, cyanobacteria, to exacerbate adverse outcomes reported in studies of spring viraemia of carp. Our study confirmed our hypothesis that environmentally relevant combined exposure (cyanobacterial biomass and viral agent) worsened markers related to the functionality of immune system and liver health. Furthermore, to the best of our knowledge, our study newly investigated the impact of naturally occurring stressors on the bioaccumulation/detoxification potency of MCs in fish. The combined exposure to cyanobacterial biomass and virus modulated detoxification processes, which may have implications in fish responses to other potential co-exposures to toxicants. The future perspective is to provide detailed insight into how other environmentally relevant stressors modulate the toxicity of cyanobacteria or the manifestation of viral diseases.

## 4. Materials and Methods

### 4.1. Animal and Experimental Design

The experiment was performed with one-year-old common carps (*Cyprinus carpio* L.). Experimental fish were obtained from the Department of Zoology, Fisheries, Hydrobiology and Apiculture of Mendel University in Brno. All experiments were performed in compliance with the law for the protection of animals against cruelty as approved by the Ethical Committee of the University of Veterinary and Pharmaceutical Sciences Brno, Czech Republic (approval no. 52/2009). Fish were distributed into four groups, each of them containing 50 individuals, and each group was kept in laminated circular tanks with their own recirculation with volume 1 m^3^ (water temperature 19.6–21.8 °C, oxygen saturation 72–80%, pH 7.35–8.16, 10 h light/14 h dark). The acclimatization lasted 14 days, and fish were fed with commercial granulated food. Ten fish from each tank were signed individually with identification chips. The initial average body weight was 352.0 ± 59.8 g, and the average total length 258.4 ± 16.1 mm. Four different exposure variants were investigated: C = Control group, B = Cyanobacterial biomass exposed group, SVC = *Carp sprivivirus* infected group, B+SVC—Combined exposure (cyanobacterial biomass and *Carp sprivivirus* infection). The fish were exposed for 42 days. The beginning of exposition to cyanobacterial biomass was assigned as day T-7 of the experiment. Exposition to cyanobacterial biomass was made perorally (groups B and B+SVC). Fish were fed the same food with the addition of 3% of lyophilized toxic cyanobacterial biomass (monoculture of *Microcystis aeruginosa*) containing 1087 μg/g MC-LR and 1462 μg/g MC-RR [39]. The whole amount of microcystins was 27 mg/1 kg of food, i.e., 0.4 mg/kg of fish weight per day. Fish from groups C and SVC were fed with commercial granulated food (Dibaq Carpio Plus, Spain). Feeding was performed 3× daily with an amount of feed equal to 1% of the weight of fish stock (1% was selected to avoid extensive growth of the fish). Adaptation of the feed ration was made weekly on the basis of actual fish weight. At day T0, seven days after the start of cyanobacterial biomass application, fish from groups SVC and B+SVC were intramuscularly injected with 0.1 mL of supernatant of SVC virus (10^2^ TCID_50_/fish) [40]. The virus was prepared according to Forlenza et al. [41]. Briefly, SVCV strain CAPM V 539 was propagated in the Epithelioma Papulosum Cyprini (EPC) cell line at 15 °C [42]. Cells were grown in Eagle’s Minimal Essential Medium (MEM, Sigma-Aldrich, USA) containing 2% fetal bovine serum, 100 IU/mL of penicillin, 100 µg/mL of sterptomycin, and 100 µg/mL of gentamycin. Sterile MEM was applied to the fish from groups C and B instead of the virus. On days T0, T14, T21, T28, and T42, eight fish from each group were euthanized, pathomorphologically examined, and individual tissues were taken for evaluation and measurements. At the end of the experiment, all remaining fish were euthanized.

### 4.2. Experimental Timepoints and Tissue Sampling

Body size measurements, pathological evaluation, sampling of liver, kidney, and gut tissue (for histopathology), blood sampling (for hematology, biochemistry, and respiratory burst measurements) and liver tissue sampling (for evaluation of microcystin and microcystin conjugates by liquid chromatography mass spectrometry) was performed at T0 (groups C and B) and T7, T14, T21, T28, and T42 (all groups).

Body weight, total length, body weight without entrails, and liver weight were recorded and the hepato-somatic index was calculated. The peripheral blood (1.5 mL) was collected by cardial puncture using the heparinized syringes, and hematological and respiratory burst measurements were performed. Moreover, 1 mL of the blood was centrifuged (400× *g*, 15 min at 4 °C); afterwards, the blood plasma was harvested and frozen immediately (−20 °C). At T14 and T42, five samples of liver, kidney, and gut from each group were collected with 10% *v*/*v* formaldehyde for histopathological analysis.

Additionally, non-heparinized blood of ten individually signed fish from each tank was collected at T0, T21, and T42, and the blood serum was harvested. The whole Ig concentration as well as specific antibodies against spring viremia carp virus were evaluated by ELISA (ELISA described in Section 4.9).

### 4.3. Body size Measurements

The following parameters were evaluated at T0, T7, T14, T21, T28, and T42: the length of the body, height and width of the body, weight of the body, weight of the body without entrails, and weight of the liver.

### 4.4. Pathological Evaluation and Histopathology

After blood samples were taken, the fish were euthanized by stunning with a blow to the back of the head followed by spinal transaction. A full necropsy was performed, and external and internal organs were assessed for macroscopic changes. Samples of the formalin-fixed kidney, liver, and gut (from 5 fish per group) were dehydrated in a series of graded ethanol, embedded in paraffin wax, and from the blocks, two consecutive 5 μm sections were cut and stained with hematoxylin and eosin.

### 4.5. Hematology

Hematological measurements were performed at T0, T7, T14, T21, T28, and T42. Hematocrit (PCV) was measured using microhematocrit tubes. Hemoglobin (Hb) was determined by the cyanmethemoglobin method. Total red blood cell (RBC) and white blood cell (WBC) counts were determined manually using a hemocytometer with Natt–Herrick’s solution. Blood smears (one slide for each fish examined) were made and stained with a Hemacolor Rapid staining kit (Merck, Darmstadt, Germany). For each smear, one hundred WBCs were counted and classified as lymphocytes, neutrophils (segmented neutrophils, bars, myelocytes/metamyelocytes), monocytes, and blast cells [43].

### 4.6. Blood Biochemistry

We centrifuged heparinized blood (400× *g*, 15 min, 4 °C), and collected plasma was stored at −80 °C. KONELAB T20xt (Thermo Fisher Scientific, Finland), a clinical analyzer, was used to analyze biochemical markers. Alanine aminotransferase (ALT) activity was determined by a method based on the kinetic assessment of NADPH consumption that generates pyruvate [44]. Lactate dehydrogenase (LDH) was measured according the method analyzing the formation of NADH during the conversion of L-lactate to pyruvate [45]. Alkaline phosphatase (ALP) was determined by a modification of the enzymatic method using an AMP (adenosine monophosphate) buffer [46].

Total serum protein (TP) was determined by the biuret reaction [47]. Albumin (ALB) was determined by the photometric method with bromocresol green [48]. Glucose (GLU) concentration was determined by the glucose hexokinase method at 37 °C [49]. Calcium (Ca) and magnesium (Mg) concentrations were determined by modified colorimetric methods with arzenazo III [50]. Phosphorus (P) was determined by an endpoint method with sample blanking using an ammonium molybdenate reagent [51]. Iron (Fe) was determined by the photometric method with ferene (ferroin-type reagent) without deproteination [52]. Urea (UREA) concentrations were determined by the kinetic enzymatic method with urease [53]. The cholesterol (CHOL) was determined by the CHOD-PAP method after enzymatic hydrolysis and oxidation [54]. The creatinine (CREA) was determined by the Jaffe kinetic method without deproteination [55].

### 4.7. Respiratory Burst Measurement

Respiratory burst measurements were performed at T0, T7, T14, T21, T28, and T42. Whole heparinized blood was used for the evaluation of phagocyte activity by luminol-enhanced chemiluminescence by using the modified method according to Kubala et al. [56].

The phagocyte activity was measured as respiratory burst activity by a method based on luminol-enhanced chemiluminescence. The reaction mixture contained 50× diluted blood in Hank’s balanced salt solution, luminol (Molecular Probes, USA) dissolved in borate buffer with Zymosan A (0.25 mg/mL), opsonized in the presence of fish serum, which was used as the activator.

The results are expressed as integrals calculated from the obtained kinetic curves and as integrals normalized per 1000 phagocytes (neutrophils + monocytes) present in the sample. The stimulation index was calculated as the ratio of opsonized zymosan-stimulated and non-stimulated blood. For more details, see the methodology in Buchtikova et al. [57].

### 4.8. Measurement of Microcystin and Microcystin Conjugates by Liquid Chromatography Mass Spectrometry Analyses

Analyses of microcystins and microcystin conjugates were performed by means of liquid chromatography and electrospray ionization mass spectrometry analyses. Briefly, analyte separation was achieved using the HPLC apparatus Agilent 1290 series (Agilent Technologies, Waldbronn, Germany) consisting of a vacuum degasser, a binary pump, an autosampler, and a thermostatted column compartment kept at 30 °C. The column was a Supelcosil ABZ+Plus RP-18 endcapped (5 μm) 150 × 4.6 mm i.d. (Supelco). A SecureGuard C18 (Phenomenex, Torrance, CA, USA) guard column was used. The mobile phase consisted of 5 mM ammonium acetate in water, pH 4 (A), and methanol–acetonitrile mixture (1:1) with 5 mM ammonium acetate (B). The binary pump gradient was linear (increase from 20% B at 0 min to 90% B at 15 min, then 90% B for 5 min, then 5 min equilibration to initial conditions); the flow rate was 0.5 mL/min. 5 μL of individual sample was injected for the analyses.

The mass spectrometer was an AB Sciex 5500 QTrap (AB Sciex, Concord, ON, Canada) with electrospray ionization (ESI). Ions were detected in the positive mode. The ionization and acquisition parameters, as well as method quantitation limits and reproducibility are presented in Table 2 and Table 3, respectively. The scheduled MRM mode was used for the separation and detection of the analytes. Quantification of analytes was based on the internal standards of the respective conjugates. More details can be found in our previous papers (Kohoutek et al., 2019) [58] and (Kohoutek et al., 2010) [59].

### 4.9. Determination of Whole Immunoglobulin Concentration and Specific Antibodies against Carp Spring Virus by ELISA

Determination of whole Ig level was performed at T0, T21, and T42 in the blood serum of ten individually signed fish from each tank. Total whole immunoglobulin levels were evaluated in serum by using ELISA. The microtiter plates (GAMA, Czech Republic) were filled with 100 μL of monoclonal antibody 1E10/2A8 against carp Ig [60] diluted 1:10,000 in bicarbonate buffer (pH 9.6) and incubated overnight at 4 °C. The wells were washed 3× with PBST solution (0.1% Tween 20 in phosphate-buffered saline (PBS) solution, pH 7.2); then, they were blocked with 100 μL of blocking solution (2% fetal bovine serum (FBS) in PBST) for 1 h at room temperature and finally washed 3× with PBST solution. Carp sera were applied to the plate in four dilutions (1:500, 1:1000, 1:2000, and 1:4000) in duplicates and incubated for 1 h at 37 °C in a humid chamber. Each plate also contained 7 dilutions of purified carp Ig at a concentration range of 0.25–15.9 µL/mL as standards. Then, the wells were washed 3× with PBST, and 50 μL of monoclonal antibody 1E10/2A8 (which was conjugated with HRP by periodate method as described by Boorsma et al. [61]) was applied to each well in dilution 1:2000. The plates were incubated for 1 h at 37 °C and washed with PBST. Then, 100 μL of TMB substrate (Test-Line, s.r.o.) was added for 10 min at room temperature. The reaction was stopped with 100 μL of 1 M sulfuric acid. Absorbance was measured at 450 nm (OD_450_) by an ELISA reader. The calibration curve was constructed from OD_450_ of standard carp Ig. Then, the concentration of carp Ig (mg/mL) was calculated by using the calibration curve.

The level of specific anti-SVCV antibodies was evaluated in the blood serum of ten individually signed fish in groups SVC and B+SVC at T0, T21, and T42 by using the ELISA method. The microtiter plates (GAMA, Czech Republic) were filled with 100 μL of viral and control antigen diluted 1:100 in bicarbonate buffer (pH 9.6) and incubated overnight at 4 °C. The viral antigen was prepared by the purification of SVCV multiplied on the *Epithelioma papulosum cyprini* (EPC) cell line at 16 °C until full cytopathic effect. Then, cell debris was removed by centrifugation at 3000× *g* for 15 min at 4 °C, and the suspension was ultracentrifuged at 76,000× *g* for 2 h at 10 °C. The resulting virus pellet was resuspended in PBS to obtain a 100× concentrate of the original volume. The control antigen was prepared analogically from the non-infected EPC cell line. The wells were washed 3× with PBST solution; then, they were blocked with 200 μL of blocking solution (5% FBS in PBST) for 30 min at room temperature and finally washed 3× with PBST solution. Carp sera were applied to the plate in four dilutions (1:50–1:400/1:6400) in duplicates (one for positive and one for negative antigen) and incubated for 1 h at 37 °C in a humid chamber. Each plate also contained SVCV-positive carp serum as a positive control. Then, the wells were washed 3× with PBST, and 100 μL of monoclonal antibody 1E10/2A8 [60] against carp Ig diluted 1:50′000 was applied to each well. The plates were incubated for 1 h at 37 °C and washed with PBST. Then, 100 μL of HRP-conjugated anti-mouse antibody (Dako) diluted 1:1000 was added, incubated for 1 h at 37 °C, and washed with PBST. Then, 100 μL of TMB substrate (Test-Line, s.r.o.) was added for 10 min at room temperature. The reaction was stopped with 100 μL of 1 M sulfuric acid. Absorbance was measured at 450 nm (OD_450_) by ELISA reader, and the results are given as the difference of values of the positive and the negative antigen (pure absorbance).

### 4.10. Statistical Evaluation

All statistical analyses were performed using Statistica for Windows^®^ 12.0 (StatSoft, Tulsa, OK, USA). The homogeneity of variance was tested by Levene’s test. In these cases, the nonparametric Kruskal–Wallis and Mann–Whitney tests were used for the comparison of treatment groups. Statistical differences within the group at different time intervals were evaluated by a regression curve. Values of *p* < 0.05 and *p* < 0.01 were considered statistically significant and highly significant, respectively, for all tests.

## Figures and Tables

**Table 1:** The biochemical indices in blood plasma of common carp [average ± SD]. C—control, B—cyanobacterial biomass exposed group, SVC—spring viremia of carp infected group, B+SVC—combined (B+SVC) group, T—days after start of the experiment. Significant differences among indices are marked by lower letters (a, b, c) for *p* < 0.05 or capital letters (A, B for *p* < 0.01. Measured parameters: ALP (alkaline phosphatase), ALT (alanine aminotransferase), LDH (lactate dehydrogenase), CHOL (cholesterol), CREA (creatine), TP (total serum protein), UREA (urea), Ca (calcium), Mg (magnesuim), and P (phosphorus). Eight fish from each group were tested at each time point.

Indices	Group	T0	T7	T14	T21	T28	T42
ALP (µkat/l)	C	0.37 ± 0.22 0.67 ± 0.55	0.69 ± 0.33	0.36 ± 0.46^ab^	0.69 ± 0.32	0.95 ± 1.26	0.56 ± 0.50
	B		0.46 ± 0.40	0.16 ± 0.16^a^	0.59 ± 0.27	0.25 ± 0.13	0.46 ± 0.42
	SVC		0.78 ± 0.34	0.93 ± 0.75 ^b^	0.56 ± 0.24	0.50 ± 0.12	0.32 ± 0.27
	B+SVC		0.62 ± 0.34	0.54 ± 0.23 ^ab^	0.74 ± 0.37	0.72 ± 0.42	0.42 ± 0.22
ALT (µkat/l)	C	0.38 ± 0.18 0.53 ± 0.32	0.24 ± 0.08	0.24 ± 0.21 ^ab^	0.22 ± 0.07 ^ab^	0.16 ± 0.05 ^a^	0.18 ± 0.09
	B		0.35 ± 0.20	0.15 ± 0.07 ^A^	0.18 ± 0.06 ^A^	0.29 ± 0.12 ^ab^	0.24 ± 0.09
	SVC		0.27 ± 0.07	0.47 ± 0.05 ^ab^	0.43 ± 0.11 ^bc^	0.33 ± 0.13 ^b^	0.19 ± 0.08
	B+SVC		0.34 ± 0.10	0.62 ± 0.24 ^B^	0.51 ± 0.19 ^C^	0.22 ± 0.07 ^ab^	0.25 ± 0.11
LDH (µkat/l)	C	25.51 ± 21.29 30.40 ± 15.25	7.43 ± 3.79 ^ab^	3.49 ± 0.58	9.48 ± 7.10	4.96 ± 3.26	6.36 ± 7.08
	B		11.72 ± 19.62 ^a^	4.55 ± 4.46	10.39 ± 11.20	11.08 ± 9.32	7.63 ± 5.13
	SVC		14.09 ± 4.58 ^b^	14.38 ± 11.96	14.48 ± 7.52	7.00 ± 7.44	4.53 ± 3.21
	B+SVC		11.82 ± 6.22 ^ab^	16.97 ± 11.36	6.41 ± 4.05	6.23 ± 4.88	7.63 ± 7.30
CHOL (mmol/l)	C	5.21 ± 1.09 5.06 ± 0.93	5.51 ± 1.10	5.34 ± 0.34 ^ab^	4.66 ± 0.49 ^a^	4.62 ± 0.45 ^A^	4.88 ± 0.52 ^A^
	B		5.63 ± 0.75	4.77 ± 0.82 ^ab^	5.43 ± 0.85 ^ab^	6.16 ± 0.67 ^B^	5.55 ± 0.73 ^ab^
	SVC		4.93 ± 0.62	4.87 ± 0.30 ^a^	5.46 ± 0.59 ^ab^	6.58 ± 0.77 ^b^	5.31 ± 0.56 ^ab^
	B+SVC		5.02 ± 0.87	5.74 ± 0.40 ^b^	6.62 ± 0.81 ^b^	4.84 ± 0.71 ^a^	6.22 ± 0.80 ^B^
CREA (µmol/l)	C	31.41 ± 13.22 33.61 ± 10.03	34.75 ± 7.72	21.58 ± 6.36 ^a^	26.52 ± 2.55	32.69 ± 3.96 ^a^	25.20 ± 4.21 ^a^
	B		37.00 ± 13.57	31.97 ± 6.97 ^ab^	24.97 ± 2.83	30.11 ± 3.95 ^ab^	31.27 ± 3.94 ^ab^
	SVC		40.41 ± 11.35	33.97 ± 4.15 ^b^	29.72 ± 3.63	29.39 ± 4.45 ^ab^	26.51 ± 4.03 ^ab^
	B+SVC		37.75 ± 20.08	31.58 ± 7.69 ^ab^	25.75 ± 3.54	25.54 ± 3.52 ^b^	32.92 ± 5.80 ^b^
TP (g/l)	C	33.31 ± 2.46 33.01 ± 3.22	33.21 ± 2.71	30.70 ± 3.27 ^ab^	28.81 ± 1.89	32.33 ± 6.38	26.32 ± 1.76 ^A^
	B		33.45 ± 3.25	31.37 ± 2.06 ^a^	29.70 ± 2.89	32.18 ± 3.11	29.30 ± 2.59 ^ab^
	SVC		31.30 ± 1.52	36.06 ± 2.20 ^b^	35.73 ± 2.97	35.52 ± 3.22	31.43 ± 1.58 ^B^
	B+SVC		35.58 ± 7.58	35.46 ± 3.96 ^ab^	36.77 ± 1.91	30.78 ± 3.64	32.72 ± 3.17 ^B^
UREA (mmol/l)	C	1.06 ± 0.32 1.72 ± 0.55	1.78 ± 0.57 ^ab^	1.49 ± 0.75 ^ab^	1.79 ± 0.66 ^ab^	2.43 ± 0.73	1.80 ± 0.59
	B		1.71 ± 0.54 ^a^	1.24 ± 0.17 ^a^	1.24 ± 0.60 ^A^	1.60 ± 0.64	1.92 ± 0.37
	SVC		2.58 ± 0.50 ^b^	3.80 ± 1.09 ^b^	2.46 ± 0.45 ^b^	1.62 ± 0.46	1.98 ± 0.71
	B+SVC		2.03 ± 0.49 ^ab^	1.89 ± 1.05 ^ab^	2.64 ± 0.63 ^B^	2.22 ± 0.73	2.17 ± 0.97
Ca (mmol/l)	C	2.38 ± 0.13 2.37 ± 0.19	2.64 ± 0.23	2.28 ± 0.24	2.36 ± 0.15 ^a^	2.61 ± 0.18	2.30 ± 0.09 ^a^
	B		2.68 ± 0.35	2.56 ± 0.53	2.38 ± 0.13 ^ab^	2.51 ± 0.15	2.43 ± 0.25 ^ab^
	SVC		2.50 ± 0.22	2.55 ± 0.19	2.81 ± 0.46 ^b^	2.53 ± 0.24	2.48 ± 0.15 ^ab^
	B+SVC		2.49 ± 0.11	2.52 ± 0.45	2.74 ± 0.28 ^b^	2.36 ± 0.17	2.67 ± 0.49 ^b^
Mg (mmol/l)	C	1.13 ± 0.14 1.03 ± 0.11	1.08 ± 0.12	1.01 ± 0.15	1.09 ± 0.10 ^a^	1.18 ± 0.20	1.09 ± 0.10 ^a^
	B		1.12 ± 0.06	0.82 ± 0.34	1.12 ± 0.11 ^ab^	1.18 ± 0.10	1.16 ± 0.10 ^ab^
	SVC		1.22 ± 0.09	1.14 ± 0.11	1.24 ± 0.14 ^b^	1.23 ± 0.11	1.30 ± 0.16 ^ab^
	B+SVC		1.18 ± 0.18	1.09 ± 0.11	1.22 ± 0.07 ^b^	1.09 ± 0.10	1.26 ± 0.13 ^b^
P (mmol/l)	C	1.36 ± 0.39 1.50 ± 0.23	1.39 ± 0.24 ^A^	1.43 ± 0.22	1.54 ± 0.38	1.26 ± 0.19	1.15 ± 0.27
	B		1.47 ± 0.46 ^A^	1.35 ± 0.27	1.41 ± 0.18	1.25 ± 0.17	1.20 ± 0.26
	SVC		1.75 ± 0.18 ^B^	1.30 ± 0.19	1.76 ± 0.24	1.28 ± 0.12	1.47 ± 0.42
	B+SVC		1.03 ± 0.38 ^ab^	1.22 ± 0.38	1.51 ± 0.37	1.18 ± 0.24	1.38 ± 0.36

**Table 2:** Instrument ionization parameters.

Ion mode	ESI
Polarity	positive
Capillary voltage (V)	5500
Drying gas temperature (°C)	350
Drying gas (GAS1, psi)	40
Nebulizer (GAS2, psi)	30
Curtain gas (psi)	15

**Table 3:** Analyte-dependent instrument acquisition parameters, analyte retention time (Rt), and method quantitation limits (MQL, based on signal-to-noise ratio S/N = 10, peak-to-peak method), recovery (%, *n* = 18), and reproducibility (given as recovery standard deviation, S.D., ± %).

Analyte	Ion	Parent (m/z)	Fragment (m/z)	Rt (min)	MQL (ng/g FW)	Recovery (%)	S.D. ( ± %)
MC-RR-Cys	[M+2H]^2+^	580.3	135.1	10.4	3.3	88.5	13.0
			102.9				
MC-RR-GSH	[M+2H]^2+^	673.2	608.9	10.2	4.2	91.5	12.0
			102.9				
MC-LR-Cys	[M+2H]^2+^	558.8	135.1	11.1	3.4	107.0	8.8
			102.9				
MC-LR-GSH	[M+2H]^2+^	651.9	135.1	10.8	5.0	100.0	10.0
			102.9				
MC-RR	[M+2H]^+^	519.8	135.2	8.4	3.6	25.0	9.0
			127.1				
MC-LR	[M+H]^+^	1045.5	135.2	10.2	16.2	25.0	10.0
			127.1				
MC-YR	[M+H]^+^	995.5	135.2	14.0	16.2	25.0	7.0
			127.1				

## Data Availability

The data presented in this study are available on request from the corresponding author.

## Supplementary Materials

The following are available online at https://www.mdpi.com/article/10.3390/toxins13090601/s1, Figure S1: Hematological parameters, Figure S2: Respiratory burst, Figure S3: Biochemical indices in blood plasma, Figure S4: Concentration of microcystins in liver.
