# Supplementary material for: Cyanobacteria Microcystis aeruginosa Contributes to the Severity of Fish Diseases: A Study on Spring Viraemia of Carp
Source: Toxins (Basel). 2021 Aug 28;13(9):601. doi: 10.3390/toxins13090601 (PMC8473110; doi:10.3390/toxins13090601)

Supplementary Materials: Cyanobacteria *Microcystis aeruginosa* Contributes to the Severity of Fish Diseases: A Study on Spring Viraemia of Carp

Miroslava Palikova, Radovan Kopp, Jiri Kohoutek, Ludek Blaha, Jan Mares, Petra Ondrackova, Ivana Papezikova, Hana Minarova, Lubomir Pojezdal and Ondrej Adamovsky

Figure S1. Hematological parameters. Blast cells, phagocytes (neutrophils + monocytes), lymphocytes, lymphocyte/phagocyte ratio, myelocytes monocytes and segmented neutrophils in fish exposed to cyanobacterial biomass, spring viremia of carp or both cyanobacterial biomass and spring viremia of carp. *Carp sprivivirus* was applied seven days after the start of cyanobacterial biomass exposition. This day was assigned as day 0 (T0). The sampling was performed at days 0, 7, 14, 21, 28 and 42 (i.e. T0, T7, T14, T21, T28, T42). Box includes the 25th to 75th percentiles, with the middle point representing the median and the spots showing the outliers. c – control, b – cyanobacterial biomass exposed group, svc – spring viremia of carp infected group, b+svc – combined exposure to cyanobacterial biomass and spring viremia of carp, T – days after start of the experiment. Significant differences among indices are marked by lower letters ($p<0.05$) or capital letters ($p<0.01$).

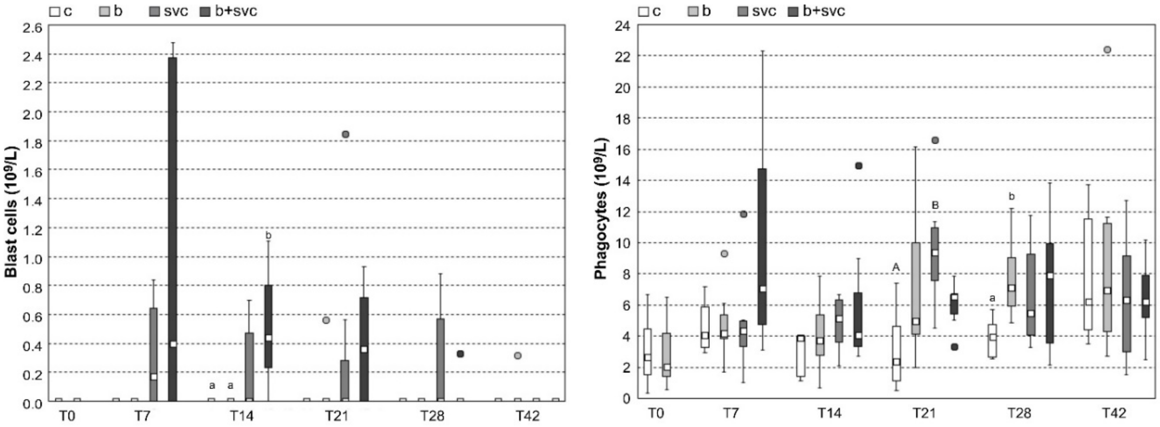

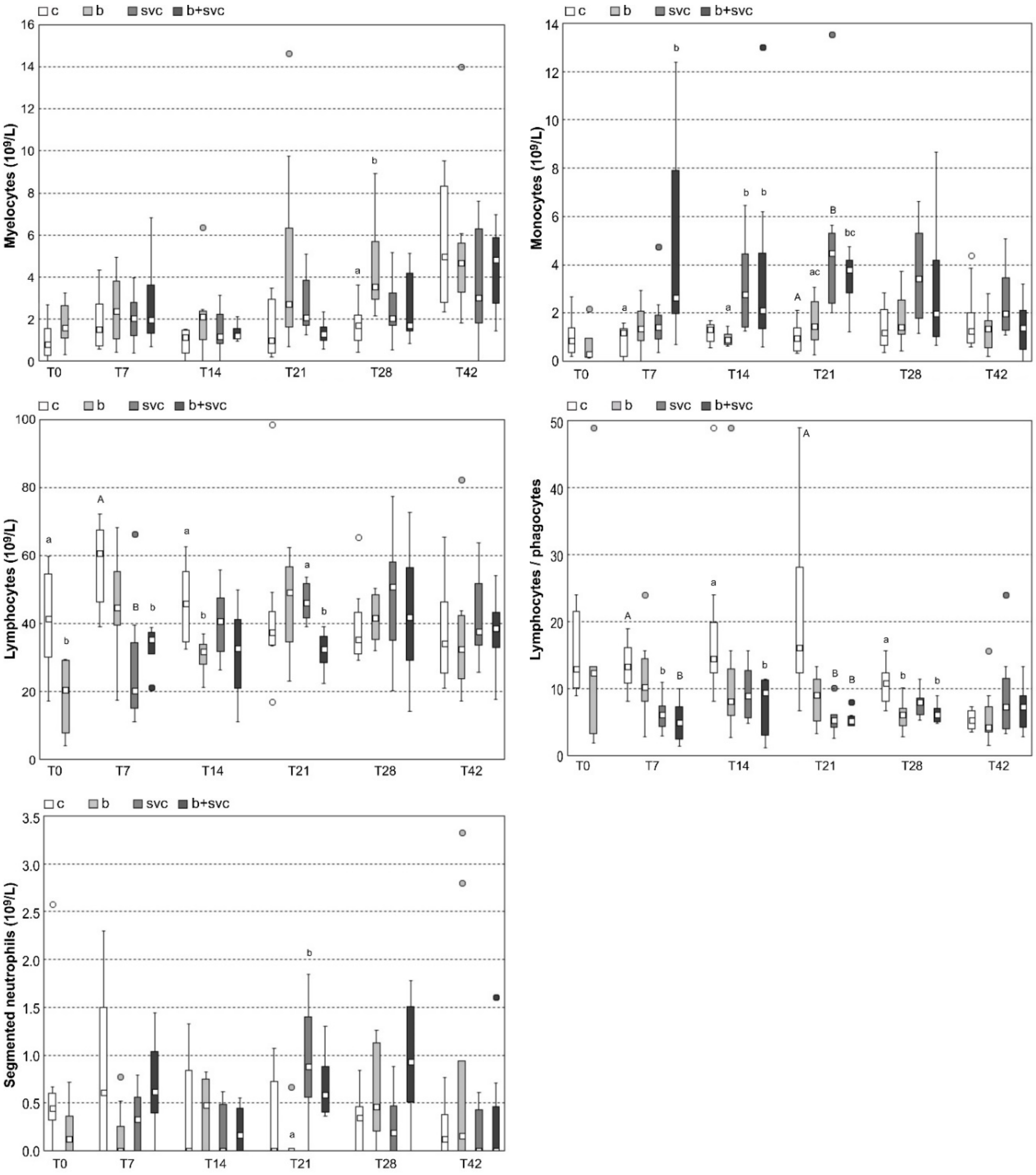

Figure S2. Respiratory burst. Chemiluminescence response of non-stimulated blood expressed in relative light units (RLU).min, and stimulation index as ratio of opsonized zymosan-stimulated and non-stimulated blood in fish exposed to cyanobacterial biomass or spring viremia of carp or both cyanobacterial biomass and spring viremia of carp. Virus of SVC was applied seven days after the start of cyanobacterial biomass exposition. This day was assigned as day 0 (T0). Box plot is 25th to 75th percentiles, with the middle point representing the median and the spots showing the outliers. Groups: C – control, B – cyanobacterial biomass exposed group, SVC – spring viremia of carp infected group, B+SVC – combined group. Significant differences among indices are marked by lower letters ($p<0.05$) or capital letters ($p<0.01$).

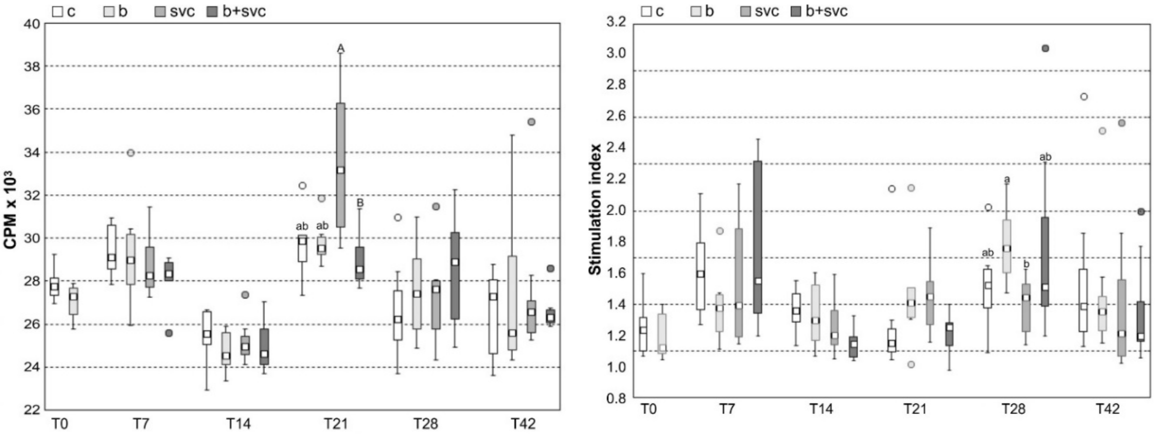

Figure S3. The biochemical indices in blood plasma of common carp [average±SD] during the studied period (eight fish from experimental groups and from control were analyzed at each sampling). Box includes the 25th to 75th percentiles, with the middle point representing the median and the spots showing the outliers. c – control, b – cyanobacterial biomass exposed group, svc – spring viremia of carp infected group, b+svc – combined (b + svc) group, T – days after start of the experiment. Significant differences among indices are marked by lower letters ($p<0.05$) or capital letters ($p<0.01$).

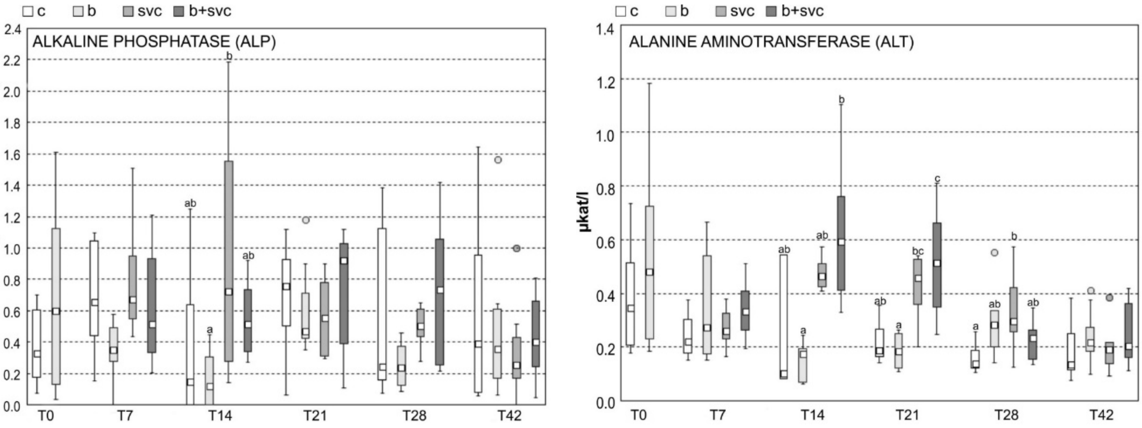

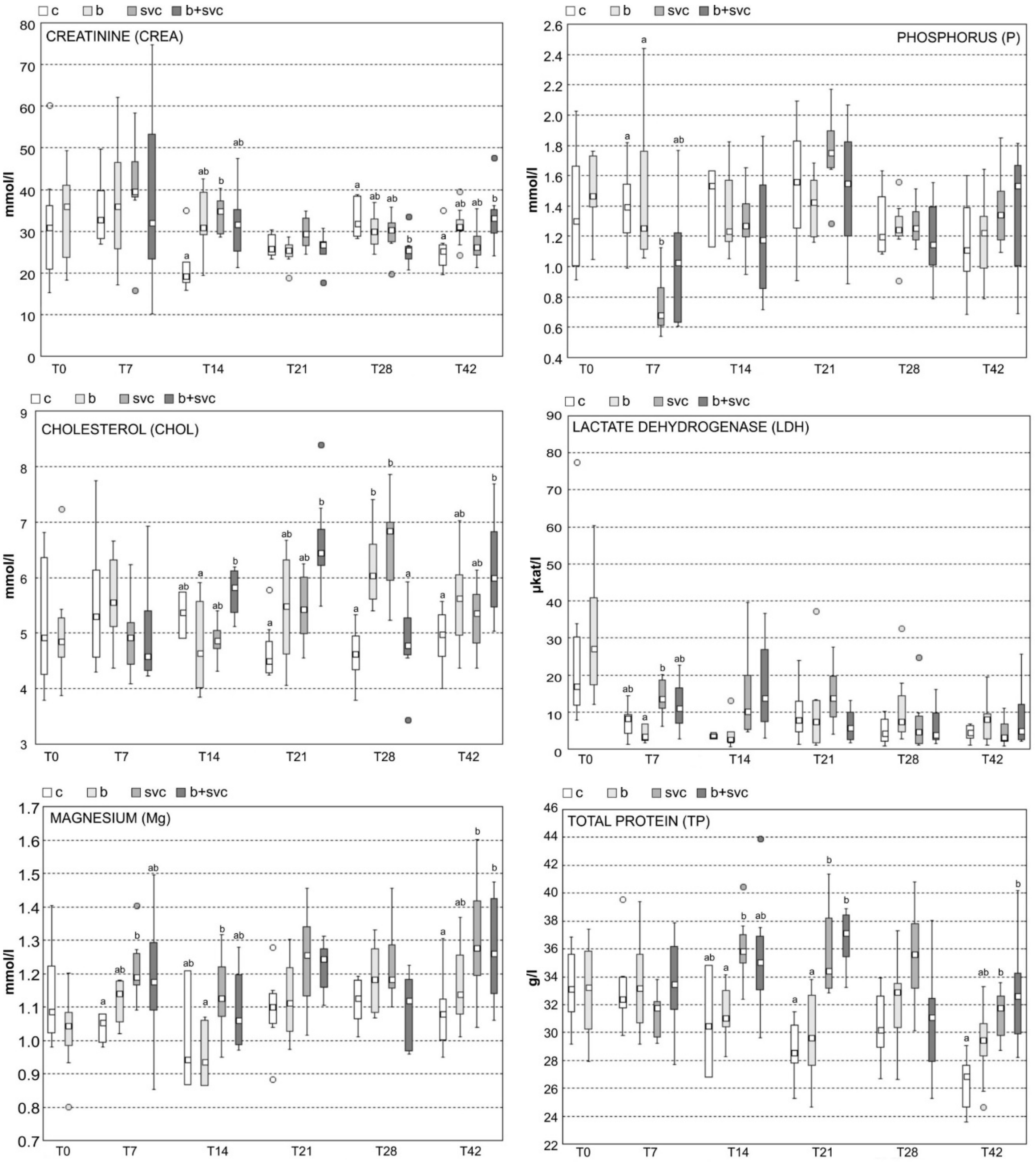
CREATININE (CREA)
mmol/l
PHOSPHORUS (P)
mmol/l
CHOLESTEROL (CHOL)
mmol/l
LACTATE DEHYDROGENASE (LDH)
µkat/l
MAGNESIUM (Mg)
mmol/l
TOTAL PROTEIN (TP)
g/l
c b svc b+svc
T0 T7 T14 T21 T28 T42

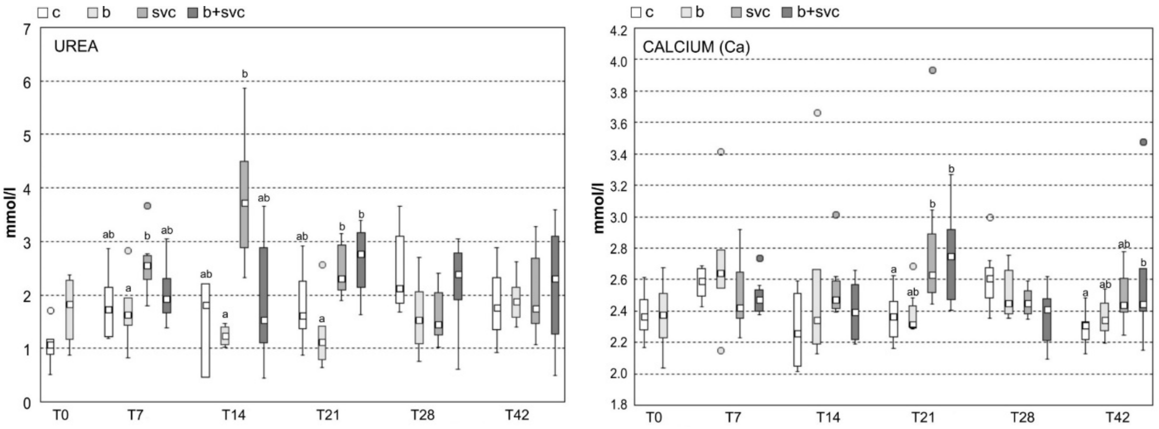

Figure S4. Concentration of microcystin-LR and –RR in fish liver. The fish were exposed to cyanobacterial biomass or both cyanobacterial biomass and spring viremia of carp. Virus of SVC was applied seven days after the start of cyanobacterial biomass exposition. This day was assigned as day 0 (T0). The sampling was performed at days 0, 7, 14, 21, 28 and 42 (i.e. T0, T7, T14, T21, T28, T42). Box includes the 25th to 75th percentiles, with the middle point representing the median, the spots showing the outliers and asterisk the extremes. b – cyanobacterial biomass exposed group, b+svc – combined (b + svc) group, T – days after start of the experiment. Statistical differences within group at time points are marked by number of day (p<0.05).

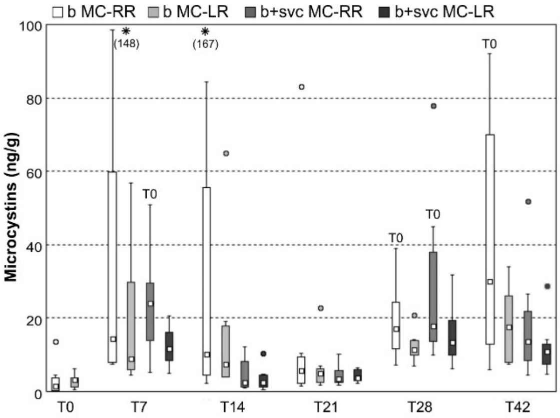

Supplement: Supplementary file 1 [file toxins-13-00601-s001.zip › toxins-1275351-supplementary.pdf]
